# 5-HT_7_ receptor modulates GABAergic transmission in the rat dorsal raphe nucleus and controls cortical release of serotonin

**DOI:** 10.3389/fncel.2015.00324

**Published:** 2015-08-18

**Authors:** Magdalena Kusek, Joanna Sowa, Katarzyna Kamińska, Krystyna Gołembiowska, Krzysztof Tokarski, Grzegorz Hess

**Affiliations:** ^1^Department of Physiology, Institute of Pharmacology, Polish Academy of SciencesKrakow, Poland; ^2^Department of Pharmacology, Institute of Pharmacology, Polish Academy of SciencesKrakow, Poland; ^3^Institute of Zoology, Jagiellonian UniversityKrakow, Poland

**Keywords:** brain slices, HPLC, microdialysis, prefrontal cortex, whole-cell recording

## Abstract

The 5-HT_7_ receptor is one of the several serotonin (5-HT) receptor subtypes that are expressed in the dorsal raphe nucleus (DRN). Some earlier findings suggested that 5-HT_7_ receptors in the DRN were localized on GABAergic interneurons modulating the activity of 5-HT projection neurons. The aim of the present study was to find out how the 5-HT_7_ receptor modulates the GABAergic synaptic input to putative 5-HT DRN neurons, and whether blockade of the 5-HT_7_ receptor would affect the release of 5-HT in the target structure. Male Wistar rats with microdialysis probes implanted in the prefrontal cortex (PFC) received injections of the 5-HT_7_ receptor antagonist (2R)-1-[(3-hydroxyphenyl)sulfonyl]-2-[2-(4-methyl-1-piperidinyl)ethyl]pyrrolidine hydrochloride (SB 269970), which induced an increase in the levels of 5-HT and its metabolite, 5-hydroxyindoleacetic acid (5-HIAA) in the PFC. In another set of experiments whole-cell recordings from presumed projection neurons were carried out using DRN slices. SB 269970 application resulted in depolarization and in an increase in the firing frequency of the cells. In order to activate 5-HT_7_ receptors, 5-carboxamidotryptamine (5-CT) was applied in the presence of N-[2-[4-(2-methoxyphenyl)-1piperazinyl]ethyl]-N-2-pyridinylcyclohexanecarboxamide (WAY100635). Hyperpolarization of cells and a decrease in the firing frequency were observed after activation of the 5-HT_7_ receptor. Blockade of 5-HT_7_ receptors caused a decrease in the mean frequency of spontaneous inhibitory postsynaptic currents (sIPSCs), while its activation induced an increase. The mechanism of these effects appears to involve tonically-active 5-HT_7_ receptors modulating firing and/or GABA release from inhibitory interneurons which regulate the activity of DRN serotonergic projection neurons.

## Introduction

The dorsal raphe nucleus (DRN) is the main source of widespread serotonin (5-hydroxytryptamine, 5-HT) projections to the forebrain, which regulate the activity of neuronal circuits involved in a spectrum of functions including emotional states, sleep, motivation and aggression (reviewed in Celada et al., 2013; Paul and Lowry, 2013). DRN 5-HT projection neurons have been thoroughly studied (reviewed in Aghajanian et al., 1990; Gaspar and Lillesaar, 2012; Andrade and Haj-Dahmane, 2013). These cells exhibit *in vivo* a regular, slow activity pattern and fire broad (2–3 ms) action potentials which are generated by a combination of voltage-dependent sodium and calcium conductances (Penington et al., 1991; Beck et al., 2004). DRN also contains numerous nonserotonergic neurons which release glutamate, GABA, as well as dopamine and peptide transmitters (Kirby et al., 2003; reviewed in Liu et al., 2000; Commons, 2009; Soiza-Reilly and Commons, 2014). Some of these cells are local interneurons (Commons, 2009; Calizo et al., 2011); however, recent work has demonstrated that many DRN projection neurons are glutamatergic or GABAergic (Jackson et al., 2009; Bang and Commons, 2012). Moreover, subsets of DRN 5-HT neurons coexpress glutamate or GABA (Commons, 2009; Shikanai et al., 2012).

The activity of DRN neurons is modulated by the locally released 5-HT (Hernandez-Lopez et al., 2013). Serotonin receptors in the central nervous system have been classified as members of seven families and at least 15 subtypes on the basis of their pharmacological properties, their coupling to intracellular signaling cascades and the protein structure (Hoyer et al., 2002). DRN 5-HT neurons have been found to express 5-HT_1A_, 5-HT_1B_, 5-HT_1D_ and possibly, 5-HT_2_ autoreceptors (McDevitt and Neumaier, 2011). Another receptor, abundant in the DRN, is the 5-HT_7_ one (Roberts et al., 2001). Beside the DRN, high levels of 5-HT_7_ receptor mRNA and protein are present in the thalamus, hippocampus, frontal cortex and hypothalamus (reviewed in Hedlund and Sutcliffe, 2004). This receptor stimulates adenylyl cyclase via G_αs_ proteins and is also coupled to the G_12_ protein; and moreover, it activates small GTPases of the Rho family (reviewed in Gellynck et al., 2013; Guseva et al., 2014). Activation of the 5-HT_7_ receptor increases the excitability of the neuron that expresses it (Bacon and Beck, 2000; Bickmeyer et al., 2002; Tokarski et al., 2003). A considerable body of experimental evidence indicates that the 5-HT_7_ receptor may be involved in the etiology of mental illnesses (reviewed in Hedlund, 2009; Ciranna and Catania, 2014). Also, recent research has suggested that the antagonists of this receptor may constitute a new class of antidepressant drugs with a faster therapeutic action than that of the currently used drugs (Mnie-Filali et al., 2011; reviewed in Tokarski et al., 2012).

It has been reported that administration of the 5-HT_7_ receptor agonist AS19 results in a reduction in the firing rate of rat DRN 5-HT cells *in vivo* (Mnie-Filali et al., 2011). On the other hand, blockade of the 5-HT_7_ receptor by the selective antagonist (2R)-1-[(3-hydroxyphenyl)sulfonyl]-2-[2-(4-methyl-1-piperidinyl)ethyl]pyrrolidine hydrochloride (SB 269970; Hagan et al., 2000) has been shown to increase the extracellular level of 5-HT in the prefrontal cortex (PFC; Wesołowska and Kowalska, 2008; but see Bonaventure et al., 2007). These findings are consistent with the hypothesis that 5-HT_7_ receptors in the DRN are not localized on 5-HT cells, but rather on local GABAergic interneurons which modulate the activity of 5-HT projection neurons (Harsing, 2006). The 5-HT_7_ receptor-dependent modulation of inhibitory influence on DRN projection neurons is has not yet been explored (Liu et al., 2000; Gocho et al., 2013; Weissbourd et al., 2014; Commons, 2015). Therefore, in the present study we aimed at determining how the 5-HT_7_ receptor activation and blockade modulate the GABAergic synaptic input to electrophysiologically identified, broad action potential-exhibiting DRN neurons in a slice preparation. We have also investigated, whether blockade of the 5-HT_7_ receptor would influence the extracellular level of 5-HT and its metabolite 5-hydroxyindoleacetic acid (5-HIAA) in the PFC *in vivo*.

## Materials and Methods

### Animals

All experimental procedures were approved by the Local Ethics Committee for Animal Experiments at the Institute of Pharmacology, Polish Academy of Sciences, and were carried out in accordance with the European Community guidelines for the use of experimental animals and the national law. Male Wistar rats (Charles River, Germany) were housed in groups in standard laboratory cages and kept in a constant temperature- and humidity-controlled colony room (21 ± 2°C) on a 12 h light/dark cycle (the light on at 07:00, off at 19:00). Commercial food and tap water were available *ad libitum*.

### Microdialysis

Rats (250–300 g) were anaesthetized with ketamine (75 mg/kg IM) and xylazine (10 mg/kg IM) and put in a stereotaxic apparatus (David Kopf Instruments, USA). The scalp was retracted and holes were drilled through the skull to insert vertical microdialysis probes into the PFC (2.9 mm anterior from the bregma, 0.8 mm lateral from the sagittal suture and −4.5 ventral from the dural surface; Paxinos and Watson, 1998). The microdialysis probes were constructed by inserting two fused silica tubes (30 and 35 mm long, 150 μm outer diameter (o.d.); Polymicro Technologies Inc., USA) into a microdialysis fiber (220 μm o.d.; AN69, Hospal, Italy). The tube assembly was placed in a stainless steel cannula (22 gauge, 10 mm) making the shaft of the probe. Parts of inlet and outlet tubes were individually placed inside the polyethylene PE-10 tubing and glued for protection. The free end of the dialysis fiber was sealed, and its 3 mm exposed length was used for a dialysis in the PFC. One day after probe implantation, the inlet of the dialysis probe was connected to a syringe pump (BAS, USA) which delivered an artificial cerebrospinal fluid (ACSF) composed of (in mM): NaCl (145), KCl (4), MgCl_2_ (1), CaCl_2_ (2.2); pH = 7.4 at a flow rate of 2.0 μl/min. After a 2 h rinsing period, baseline samples (3–4) were collected every 20 min. SB 269970 (1.25 or 2.5 mg/kg) was dissolved in a 0.9% saline and injected in a volume of 4 ml/kg IP. Samples were collected every 20 min for 4 h. At the end of the experiment the rats were sacrificed and their brains were histologically examined to validate the correct probe placement.

5-HT and 5-HIAA levels were analyzed by HPLC with a coulochemical detection. Chromatography was performed using the Ultimate 3000 System (Dionex, USA), the coulochemical detector Coulochem III (model 5300, ESA, USA) with the 5020 guard cell, the 5014B microdialysis cell and the Hypersil Gold- C_18_ analytical column (3 × 100 mm). The mobile phase was composed of 0.05 M potassium phosphate buffer adjusted to pH = 3.9, 0.5 mM EDTA, 13 mg/L 1-octanesulfonic acid sodium salt, a 3.1% methanol and a 0.93% acetonitrile. The flow rate during the analysis was 0.7 ml/min. The applied potential of a guard cell was +600 mV, while those of a microdialysis cell were E_1_ = −50 mV and E_2_ = +300 mV and a sensitivity was set at 50 nA/V. The chromatographic data were processed by the Chromeleon v. 6.80 (Dionex, USA) software run on a PC computer. The values were not corrected for an *in vitro* probe recovery which was ca. 15%.

### Slice Preparation and Incubation

Rats (150 g) were decapitated under isoflurane anesthesia (Aerrane, Baxter, UK), their brains were quickly removed and placed in an ice-cold modified ACSF containing (in mM): NaCl (130), KCl (5), CaCl_2_ (2.5), MgSO_4_ (1.3), KH_2_PO_4_ (1.25), NaHCO_3_ (26) and D-glucose (10), bubbled with the mixture of 95% O_2_–5% CO_2_. Coronal midbrain slices containing the DRN (300 μm thick) were cut using a vibrating microtome (VT1000, Leica, Germany). Two slices were obtained from one animal. Slices were then stored submerged in ACSF at 30 ± 0.5°C.

An individual slice was placed in the recording chamber, being superfused at 3 ml/min with warm (32 ± 0.5°C), modified ACSF of the following composition (in mM): NaCl (132), KCl (2), KH_2_PO_4_ (1.25), NaHCO_3_ (26), MgSO_4_ (1.3), CaCl_2_ (2.5), D-glucose (10), bubbled with 95% O_2_–5% CO_2_. Neurons were visualized using the Axioskop 2 (Zeiss, Germany) upright microscope with the Nomarski optics, a 40× water immersion lens and an infrared camera (Tokarski et al., 2007).

### Analysis of Intrinsic Excitability and Inhibitory Postsynaptic Currents

Patch pipettes were pulled from borosilicate glass capillaries using the Sutter Instrument P97 puller. The pipette solution contained (in mM): K-gluconate (130), NaCl (5), CaCl_2_ (0.3), MgCl_2_ (2), HEPES (10), Na_2_-ATP (5), Na-GTP (0.4) and EGTA (1). Osmolarity and pH were adjusted to 290 mOsm and 7.2, respectively. Pipettes had an open tip resistance of approximately 6 MΩ.

**Figure 1:**
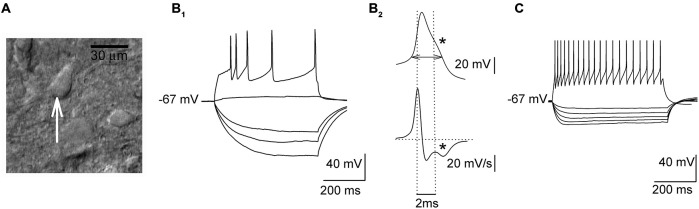
**Electrophysiological properties of sampled dorsal raphe nucleus (DRN) neurons. (A)** An image of a DRN slice (DIC optics) showing a presumed projection cell (an arrow) before the start of the recording. **(B_1_)** Responses of a presumed projection neuron to a series of hyper- and depolarizing current pulses. **(B_2_)** *Upper trace*, a single action potential shown at an expanded timescale with a “notch” (indicated with an asterisk) on the descending phase. A horizontal double arrow indicates the way the action potential width was measured. *Lower trace*, the first derivative of action potential waveform emphasizes a “notch” (an asterisk). **(C)** Responses of a presumed non-5-HT interneuron to a series of hyper- and depolarizing current pulses.

Whole-cell recordings were obtained from presumed 5-HT DRN neurons which were identified on the basis of their response to hyper- and depolarizing current pulses (Figures 1A,B; cf. Galindo-Charles et al., 2008). Cells were sampled from the dorsal part of the midline region of the DRN. Signals were recorded using the SEC 05LX amplifier (NPI, Germany), filtered at 2 kHz and digitized at 20 kHz using the Digidata 1440 interface and pClamp 10 software (Molecular Devices, USA). An input-output relationship was assessed in the current-clamp mode using hyper- and depolarizing current pulses (500 ms). To assess the relationship between the injected current and the spiking rate (gain) depolarizing current was increased in 20 pA steps, delivered every 2 s. The width of the action potential was measured at the threshold, which was determined according to Henze and Buzsáki (2001).

To record spontaneous inhibitory postsynaptic currents (sIPSCs), neurons were voltage-clamped at 0 mV (Tokarski et al., 2007). After 15 min of the stabilization period the baseline sIPSCs activity was recorded for 4 min. To block the 5-HT_7_ receptor, its selective antagonist SB 269970 (Hagan et al., 2000) was then added to the ASCF and after next 15 min of stabilization sIPSCs were recorded for 4 min. In the experiments involving activation of the 5-HT_7_ receptor slices were incubated in the ACSF supplemented with N-[2-[4-(2-methoxyphenyl)-1piperazinyl]ethyl]-N-2-pyrid inylcyclohexanecarboxamide (WAY100635, 2 μM, a selective 5-HT_1A_ antagonist; Mundey et al., 1996; Tokarski et al., 2003). After obtaining whole-cell configuration, the stabilization period (15 min) and the baseline recording (4 min), 200 nM 5-CT (a nonselective agonist) was added to the ACSF. After the following 15 min of stabilization sIPSCs were recorded for 4 min. Data were accepted for analysis when the access resistance ranged between 15 and 18 MΩ and it was stable during recordings. The recordings were inspected off-line using the Mini Analysis software (Synaptosoft) and individual synaptic events were selected manually for further analysis.

### Statistical Analysis

The results are expressed as the mean ± SEM unless otherwise noted. The statistical significance of the microdialysis data was calculated using repeated-measures ANOVA, followed by Tukey’s *post hoc* test, if applicable. Statistical analyses of the electrophysiological data were carried out using paired Student’s *t*-tests, Wilcoxon signed-rank test and Kolmogorov-Smirnov test, where applicable.

## Results

### The 5-HT_7_ Receptor Antagonist SB 269970 Induces 5-HT Release in the Prefrontal Cortex *In Vivo*

**Figure 2:**
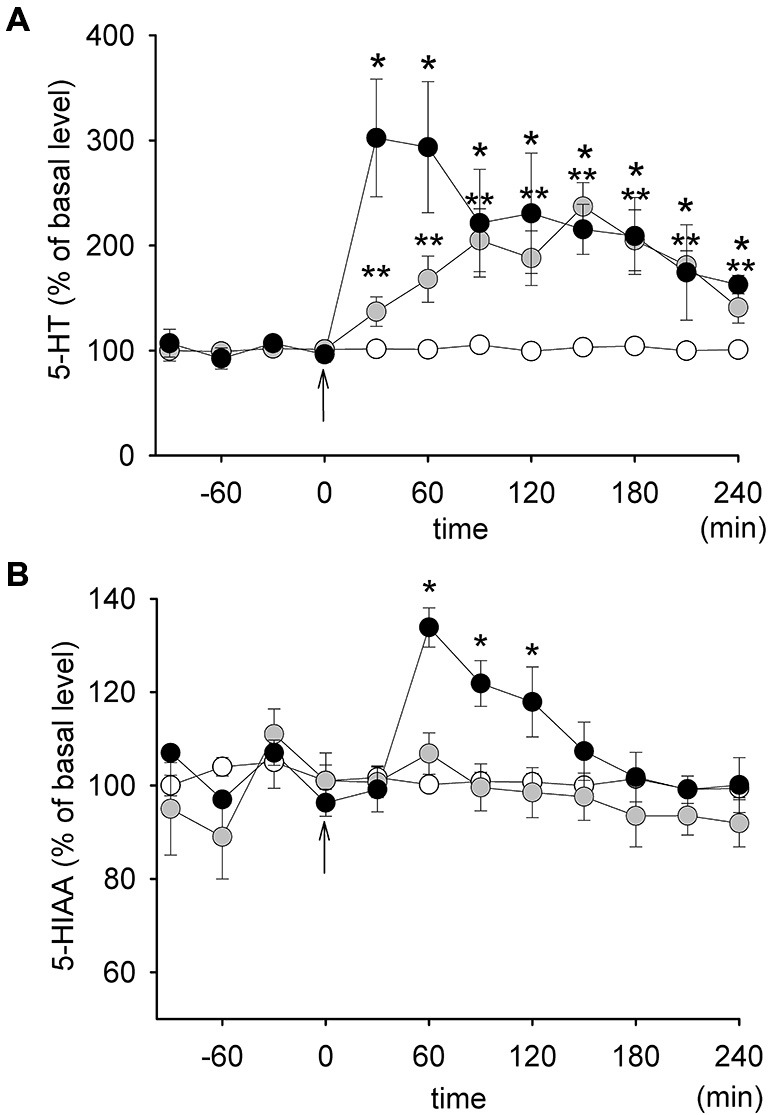
**The effect of SB 269970 on the extracellular levels of 5-HT (A) and 5-HIAA (B) in the prefrontal cortex (PFC) of freely moving rats**. Graphs represent plots of the mean normalized values (± SEM). Gray circles: 1.25 mg/kg SB 269970 (*n* = 5); black circles: 2.5 mg/kg SB 269970 (*n* = 6); open circles: control animals receiving saline (*n* = 6). *p < 0.05; **p < 0.01. Time 0 corresponds to the time of injection (an arrow).

The basal concentrations of 5-HT in dialysates from the PFC were 1.29 ± 0.19 pg/10 μl, 1.18 ± 0.16 pg/10 μl and 0.91 ± 0.09 pg/10 μl in the control group, in rats receiving 1.25 mg/kg SB 269970 and in rats receiving 2.5 mg/kg SB 269970, respectively. Those differences were not statistically significant. Injections of SB 269970 increased the extracellular level of 5-HT, which reached approximately 300% of the basal level at the higher dose of the drug during the first hour after its injection (Figure 2A). No difference in 5-HT level increase between both doses of SB 269970 was observed between 90–240 min after the administration of the drug. A significant effect of treatment with SB 269970 on 5-HT level was observed (ANOVA, *F*_(2,17)_ = 12.18, *p* < 0.0005). There was no effect of time (ANOVA, *F*_(7,119)_ = 1.16, *p* < 0.33) and there was no interaction between both factors (ANOVA, *F*_(14,119)_ = 1.59, *p* < 0.09).

The basal concentrations of 5-HIAA were 677 ± 22 pg/10 μl, 600 ± 37 pg/10 μl and 607 ± 30 pg/10 μl in the control group, in rats receiving 1.25 mg/kg SB 269970 and in rats receiving 2.5 mg/kg SB 269970, respectively. There were no significant differences between the groups. SB 269970 increased the extracellular level of 5-HIAA only at the higher dose with the maximum reaching approximately 138% of the basal level 60 min after administration (Figure 2B). This effect corresponded to an increase in the extracellular level of 5-HT but it occurred about 30 min later. A significant effect of treatment with the drug on 5-HIAA (ANOVA, *F*_(2,17)_ = 7.56, *p* < 0.004), as well as a significant effect of time (ANOVA, *F*_(7,119)_ = 14.73, *p* < 0.0001) and an interaction between both those factors (ANOVA, *F*_(14,119)_ = 8.00. *p* < 0.0001) were observed.

### The 5-HT_7_ Receptor Antagonist SB 269970 Enhances Spiking Activity and Reduces the Inhibitory Input to Neurons in DRN Slices

In response to depolarizing current pulses (500 ms), all the cells subjected to the analysis showed adaptation of the firing frequency (Figure 1B_1_). In the case of stronger current pulses (500–600 pA), an average firing frequency reached 12–16 Hz. Their action potentials were broad (3.66 ± 0.06 ms) and they showed a characteristic inflection during the descending phase of the spike (Figure 1B_2_). The mean input resistance of those cells was 432.13 ± 23.55 MΩ, and they showed no spontaneous spiking activity at the resting membrane potential (−64.5 ± 3.4 mV). As illustrated in Figure 1C, presumed non-5-HT DRN neurons exhibited higher firing frequencies (range: 36–42 Hz) and lower input resistance (260.12 ± 20.32 MΩ) than presumed 5-HT cells (cf. Galindo-Charles et al., 2008).

**Figure 3:**
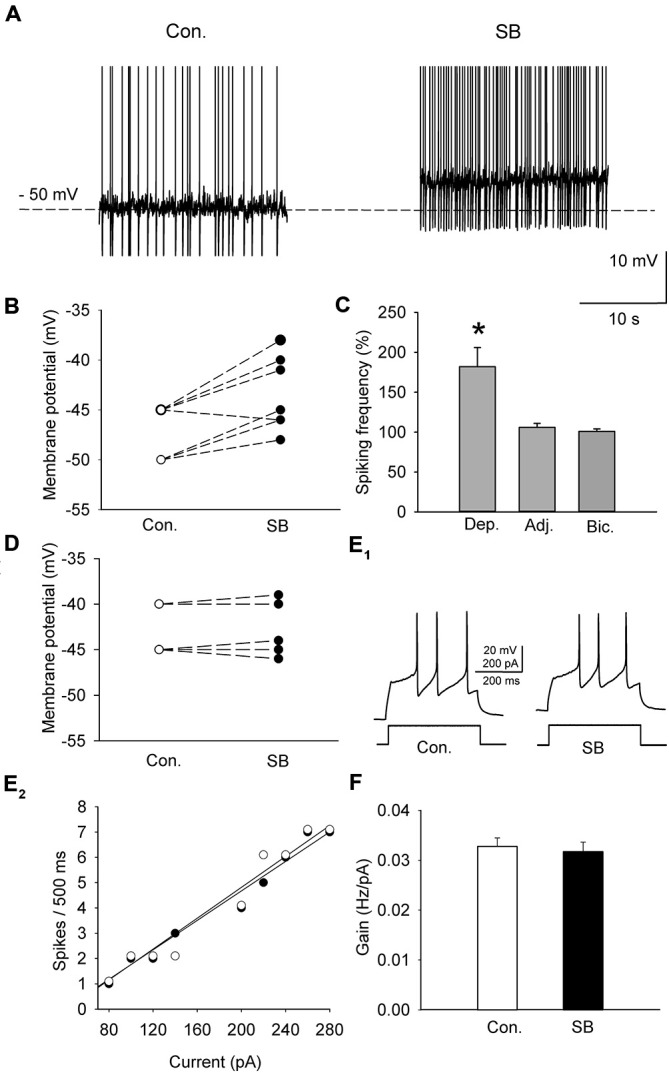
**The effect of SB 269970 (2.5 μM) on the spiking activity of DRN neurons. (A)** A representative example of the sustained spiking activity of a cell (previously depolarized to −50 mV) before (Con.) and 15 min after the addition of SB 269970 (SB) to the artificial cerebrospinal fluid (ACSF). Action potentials are truncated. **(B)** SB 269970-induced changes in the membrane potential. Neurons were initially depolarized to −50 or −45 mV to induce sustained firing. **(C)** An increase in the spiking frequency is absent after adjustment of the membrane potential of a neuron to the baseline values to prevent SB 269970-induced depolarization and in the presence of bicuculline. Shown are mean values (± SEM) of changes in the spiking frequency of cells depolarized (Dep., *n* = 11) by SB 269970, in a sample of cells whose membrane potential was adjusted (Adj., *n* = 9) to the baseline values and in a sample of cells (*n* = 5) recorded in the presence of 10 μM bicuculline (Bic.) and 2 mM kynurenic acid. **(D)** No significant change in the membrane potential was found when SB 269970 was applied in the presence of 10 μM bicuculline (Bic.) and 2 mM kynurenic acid (*n* = 5). **(E_1_)** Responses of a representative cell to a depolarizing current pulse before (Con.) and after addition of SB 269970 (SB). **(E_2_)** The relationship between the injected current vs. the spiking rate of the cell shown in **(E_1_)** before (*white circles*) and after (*black circles*) addition of SB 269970. The lines represent a linear regression. **(F)** A comparison of the relationship between the injected current and the firing rate (gain). Mean values ± SEM are shown. *p < 0.001.

A sample of cells (*n* = 11; 6 animals) was depolarized in the current-clamp mode to either −50 or −45 mV by current injection (15–55 pA) to induce sustained firing (Figure 3A). In order to study the effect of 5-HT_7_ receptor blockade on depolarization-induced sustained activity, SB 269970 (2.5 μM) was added to the ACSF, which resulted in further depolarization of the cells by 4.9 ± 0.5 mV (*n* = 11; 6 animals, paired *t-test*, *t* = −5.012, df = 10, *p* < 0.001; Figures 3A,B) and in an increase in the sustained firing frequency up to 182 ± 24% of the baseline (*n* = 11; 6 animals, paired *t-test*, *t* = −4.418, df = 10, *p* = 0.002; Figure 3C). The blockade of 5-HT_7_ receptors did not modify the excitability of DRN neurons (*n* = 6; 3 animals, paired *t-test*, *t* = 0.702, df = 5, *p* = 0.514; Figures 3E,F). No change in the sustained spiking frequency occurred when the membrane potential was adjusted to the baseline level with a steady current injection (*n* = 9; 5 animals, paired *t-test*, *t* = −1.411, df = 8, *p* = 0.196; Figure 3C). No change in the sustained spiking frequency and no depolarization occurred when SB 269970 (2.5 μM) was added to the ACSF supplemented with 10 μM bicuculline to block GABA_A_ receptors and with 2 mM kynurenic acid to block the excitatory transmission (Figures 3C,D; *n* = 5; 3 animals; spiking frequency: paired *t-test*, *t* = −1.5, df = 4, *p* = 0.208; membrane potential: paired *t*-test, *t* = −0.535 df = 4, *p* = 0.621).

**Figure 4:**
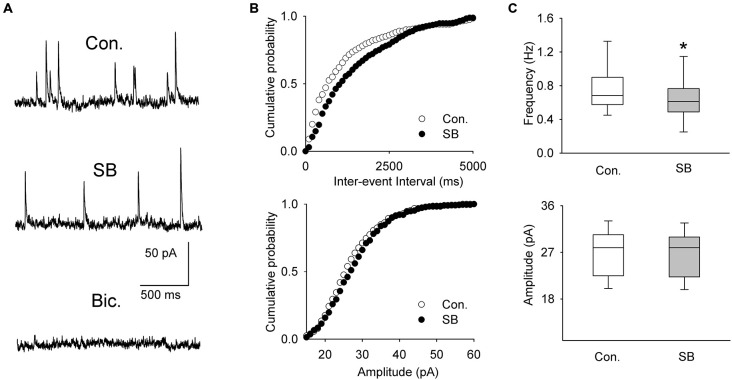
**The effect of SB 269970 (2.5 μM) on spontaneous IPSCs. (A)** Sample recordings from a representative neuron before (Con.) and 15 min after SB 269970 (SB) addition to the ACSF. Spontaneous events were blocked after addition of bicuculline methiodide (Bic., 10 μM). **(B)** Cumulative probability plots of inter-event interval (*upper graph*) and amplitude (*lower graph*) of sIPSCs recorded from a representative cell before (Con.) and after (SB) addition of SB 269970. The difference between the distributions of inter-event interval, but not amplitude, is significant (*p* = 0.0003 vs. *p* = 0.548, respectively, Kolmogorov-Smirnov test). **(C)** SB 269970—induced a small but significant decrease in the frequency (*upper graph*, *p < 0.001; Wilcoxon signed-rank test) but not amplitude of sIPSCs (*lower graph*, *p* = 0.07; Wilcoxon signed-rank test).

A separate sample of presumed DRN projection neurons (*n* = 11, 6 animals) was voltage-clamped at 0 mV to record spontaneous IPSCs (Figure 4A). The mean frequency and mean amplitude (± SEM) of sIPSCs during baseline recordings were 0.751 ± 0.084 Hz and 26.89 ± 1.36 pA, respectively. Addition of SB 269970 (2.5 μM) to the ACSF resulted in a decrease in the mean frequency of sIPSCs to 0.663 ± 0.0821 Hz (Wilcoxon signed-rank test, *p* < 0.001; Figures 4B,C). The mean amplitude of sIPSCs remained unchanged (Wilcoxon signed-rank test, 26.66 ± 1.33 pA; *p* = 0.07; Figure 4C).

### Activation of the 5-HT_7_ Receptor Suppresses Spiking Activity and Enhances the Inhibitory Input to Neurons in DRN Slices

**Figure 5:**
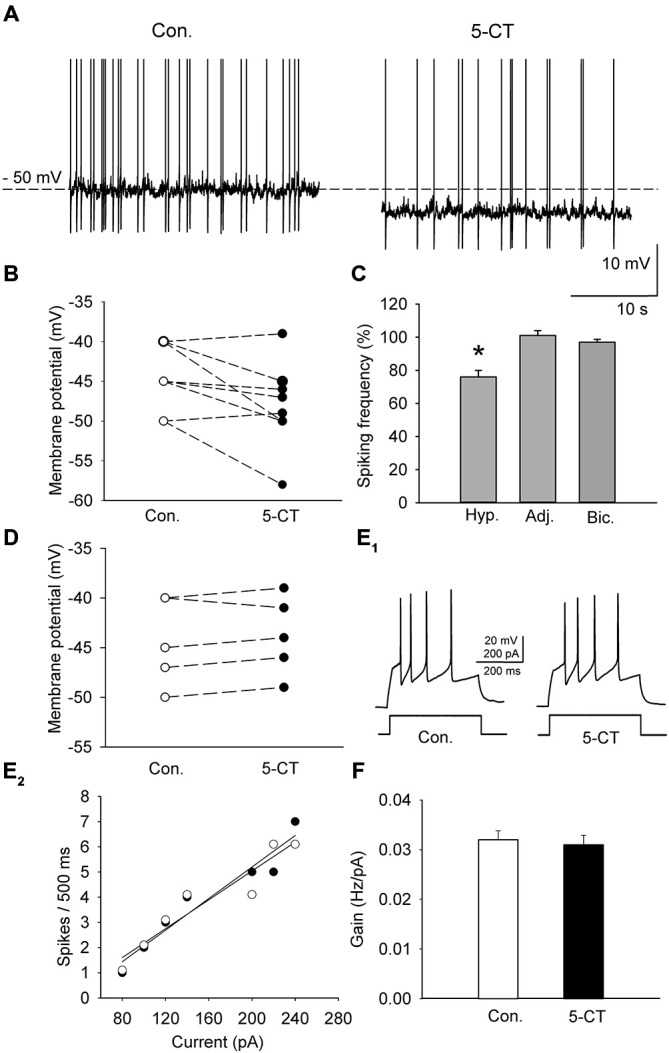
**The effect of 5-CT (250 nM), applied in the presence of WAY100635 (2 μM) on the spiking activity of DRN neurons. (A)** An example of the sustained spiking activity of a cell (previously depolarized to −50 mV) before (Con.) and 15 min after addition of 5-CT to the ACSF. Action potentials are truncated. **(B)** The 5-CT—induced changes in the membrane potential of the investigated sample of cells. Neurons were initially depolarized to induce sustained firing. **(C)** A decrease in the spiking frequency is absent if the membrane potential of a cell is adjusted to the baseline values to prevent hyperpolarization. Mean (± SEM) changes in the spiking frequency of cells hyperpolarized (Hyp., *n* = 10) by 5-CT, in a sample of cells whose membrane potential was adjusted (Adj., *n* = 8) and in a sample of cells (*n* = 5) recorded in the presence of 10 μM bicuculline (Bic.) and 2 mM kynurenic acid. **(D)** No significant change in the membrane potential was found when 5-CT was applied in the presence of 10 μM bicuculline (Bic.) and 2 mM kynurenic acid. **(E_1_)** Responses of a representative neuron to a depolarizing current pulse before (Con.) and after addition of 5-CT (5-CT). (**E_2_)** The relationship between the injected current vs. the spiking rate of the cell shown in **(E_1_)** before (white circles) and after (black circles) addition of 5-CT. The lines represent a linear regression. **(F)** A comparison of the relationship between the injected current and the firing rate (gain). Mean values ± SEM are shown. *p < 0.01.

A group of cells (*n* = 10; 5 animals) was depolarized in the current-clamp mode by current injection (25–40 pA) to induce sustained firing (Figure 5A). To activate the 5-HT_7_ receptor, 250 nM 5-CT (a nonselective agonist) was added to the ACSF supplemented with N-[2-[4-(2-methoxyphenyl)-1piperazinyl]ethyl]-N-2- pyridinylcyclohexane carboxamide (WAY100635, 2μM, a selective 5-HT_1A_ antagonist; Tokarski et al., 2003). Hyperpolarization of the cells by 4.4 ± 1.1 mV (*n* = 10; 5 animals; paired *t*-test, *t* = 2.857 df = 9, *p* = 0.019; Figures 5A,B) and a decrease in the sustained firing frequency to 76.5 ± 3.9% of the baseline (*n* = 10; 5 animals, paired *t*-test, *t* = 5.432, df = 9, *p* = 0.002; Figure 5C) were observed. Activation of the 5-HT_7_ receptor did not modify the excitability of DRN neurons (*n* = 8; 4 animals; paired *t*-test, *t* = 1.09, df = 7, *p* = 0.312; Figures 5E,F). No change in the sustained spiking frequency occurred when the membrane potential was adjusted to the baseline level with a steady current injection (*n* = 8; 4 animals, paired *t*-test, *t* = −1.83, df = 7, *p* = 0.11; Figure 5C). No change in the sustained spiking frequency and no depolarization occurred when 5-CT (250 nM) was added to the ACSF supplemented with 10 mM bicuculline to block GABA_A_ receptors and with 2 mM kynurenic acid to block the excitatory transmission (Figures 5C,D; *n* = 5; 3 animals; spiking frequency: paired *t*-test, *t* = 1.195, df = 4, *p* = 0.298; membrane potential: paired *t*-test, *t* = 0.667, df = 4, *p* = 0.541).

**Figure 6:**
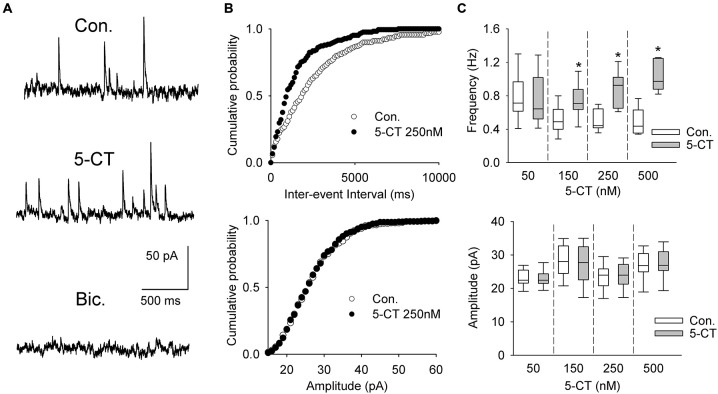
**The effect of 5-CT on spontaneous IPSCs. (A)** Recordings from a representative neuron before (Con) and 15 min after addition of 250 nM 5-CT (in the presence of 2 μM WAY100635) to the ACSF. Spontaneous events were blocked after addition of bicuculline methiodide (Bic, 10 μM). **(B)** Cumulative probability plots of inter-event interval (*upper graph*) and amplitude (*lower graph*) of sIPSCs recorded from a representative cell before (Con.) and after (SB) addition of 250 nM 5-CT. The difference between the distributions of inter-event interval, but not amplitude, is significant (*p* < 0.001 vs. *p* = 0.412, respectively, Kolmogorov-Smirnov test). **(C)** 5-CT (150–500 nM) induced an increase in the frequency (*upper graph*) but not amplitude of sIPSCs (*lower graph*). *p < 0.005; Wilcoxon signed-rank test.

A separate sample of presumed DRN projection neurons (*n* = 33, 17 animals) was voltage-clamped at 0 mV to record sIPSCs (Figure 6A). As shown in Figures 6B,C and Table 1, addition of 5-CT (50–500 nM) to the ACSF resulted in a dose-dependent increase in the mean frequency of sIPSCs.

**Table 1:** **The effect of 5-CT on spontaneous IPSCs**.

5-CT concentration (nM)	Mean control frequency (Hz)	Mean frequency in 5-CT (Hz)	Mean control amplitude (pA)	Mean amplitude in 5-CT (pA)	Number of cells
50	0.72 ± 0.15	0.69 ± 0.16	26.90 ± 1.90	26.53 ± 1.93	6
150	0.60 ± 0.14	0.75 ± 0.15*	28.22 ± 1.37	28.56 ± 1.41	13
250	0.64 ± 0.15	1.03 ± 0.17*	24.23 ± 1.56	24.34 ± 1.52	8
500	0.52 ± 0.07	1.00 ± 0.11*	28.91 ± 1.76	29.61 ± 2.30	6

Data were acquired before (control) and 15 min after addition of 5-CT (in the presence of 2 μM WAY100635) to the ACSF. *p < 0.005. Wilcoxon signed-rank test.

## Discussion

The results of the present study indicate that blockade of the 5-HT_7_ receptor enhances the release and metabolism of 5-HT in the PFC. This effect appears to be mediated by the depolarization and enhanced firing of DRN serotonergic projection neurons, resulting from a decreased inhibitory synaptic input received by the projection cells. In contrast, activation of the 5-HT_7_ receptor increases the frequency of spontaneous IPSCs, which results in hyperpolarization and reduced firing of the putative DRN projection cells.

The present results confirm and extend an earlier report that showed that blockade of the 5-HT_7_ receptor with low doses of SB 269970 (0.625 and 1.25 mg/kg) resulted in an increase in the level of extracellular 5-HT (Wesołowska and Kowalska, 2008). The effect of a higher dose of SB 269970 (2.5 mg/kg), used in the present study, was more potent, but only during the first hour after the administration. This result points to the activation of mechanisms which do not allow for an excessive tonic release of 5-HT from cortical terminals. An increase in the level of 5-HIAA is consistent with an increased level of 5-HT in the extracellular space. Another study demonstrated that SB 269970 administered in a dose of 10 mg/kg did not increase the extracellular concentration of 5-HT in the rat frontal cortex (Bonaventure et al., 2007). A most likely explanation of such an apparent discrepancy is the way of drug administration: intraperitoneal (Wesołowska and Kowalska, 2008 and our study) vs. subcutaneous (Bonaventure et al., 2007).

All the examined cells showed a low frequency, rhythmic activity after moderate depolarization, broad action potentials, adaptation of the firing frequency during depolarizing steps and an inflection on the descending phase of the spike (cf. Galindo-Charles et al., 2008). Those features were originally considered to be unique to DRN 5-HT neurons (Aghajanian et al., 1990). Later on it was shown that the population of DRN neurons with similar characteristics included non-serotonergic cells; however, there may be subtle differences between these neurons regarding e.g., the timecourse of the afterhyperpolarization (Kirby et al., 2003). We did not carry out an immunohistochemical analysis of the recorded cells, however, other investigators found that 86% of the population of identified, 5-HT-immunoreactive DRN neurons exhibited the inflection on the action potential descending phase and other features characteristic of the cell type we recorded from (Galindo-Charles et al., 2008). Some 5-HT cells also coexpress the GABAergic marker GAD67 (Shikanai et al., 2012), but “pure” DRN GABAergic interneurons, that do not synthesize 5-HT show different activity patterns, including short spikes and considerably higher discharge frequencies (Galindo-Charles et al., 2008; Shikanai et al., 2012; Gocho et al., 2013). Therefore it may be assumed that a significant fraction of the cells recorded throughout the present study represented 5-HT projection cells. The excitability of these neurons was not directly influenced by the activation or blockade of the 5-HT_7_ receptor.

It has been established that DRN neurons receive GABAergic inputs from extrinsic sources, including the hypothalamus, substantia nigra, ventral tegmental area and rostromedial tegmental nucleus (reviewed in Soiza-Reilly and Commons, 2011). Since the axons from these structures are cut during slice preparation, their contribution to the observed effects seems unlikely.

5-HT had previously been shown to induce a concentration-dependent increase in the frequency of sIPSCs recorded from the putative serotonergic neurons in DRN slices (Liu et al., 2000). The latter effect was shown to be mediated by local GABAergic interneurons, since it was blocked by the inhibitor of voltage-gated sodium channels tetrodotoxin. The stimulatory effect of 5-HT on GABAergic interneurons was attributed to the activation of 5-HT_2A_ and 5-HT_2C_ receptors; however, even high concentrations of the antagonist of those receptors did not completely block the effects of 5-HT application (Liu et al., 2000; Gocho et al., 2013).

To activate the 5-HT_7_ receptor we have applied a nonselective agonist, 5-CT, in the presence of WAY100636, a selective antagonist of the 5-HT_1A_ receptor (Mundey et al., 1996). 5-CT has been reported to be an agonist of 5-HT_1A_, 5-HT_1B_, 5-HT_1D_ and 5-HT_7_ receptors. However, the affinity of 5-CT to the 5-HT_7_ receptor is one order of magnitude higher than to the 5-HT_1B_ and 5-HT_1D_ receptors.^1^ Moreover, activation of the 5-HT_1B_ receptor has been reported to increase the firing of DRN 5-HT neurons via a reduction of the inhibitory inputs (Adell et al., 2001). Thus, the effects of 5-CT observed in the present study (increased sIPSCs frequency, hyperpolarization and reduced firing of presumable 5-HT cells) is consistent with the activation of the 5-HT_7_ receptor located on GABAergic interneurons. This conclusion is supported by the observation that blockade of the 5-HT_7_ receptor induces opposite phenomena. The observed effects of application of the 5-HT_7_ receptor antagonist indicate that the receptors are tonically active most likely due to locally released 5-HT. We showed previously that activation of 5-HT_7_ receptors resulted in the enhancement of GABAergic transmission in the hippocampal CA1 area, at least partially via 5-HT_7_ receptors located on inhibitory interneurons (Tokarski et al., 2011).

The 5-HT_7_ receptor is implicated in a wide range of pathological processes; in particular, its selective blockade has been shown to induce antidepressant-like effects in animal models (reviewed in Ciranna and Catania, 2014; Nikiforuk, 2015). The above data point to a plausible cellular mechanism of the antidepressant action of 5-HT_7_ receptor antagonists.

## Author Contributions

GH, KT and KG conceived and designed the experiments. MK, JS and KK performed the experiments. MK, JS, KG, KK, KT and GH analyzed and interpreted the obtained data. MK, JS, KG, KT and GH wrote the paper. KK helped with the manuscript preparation. MK, JS, KK, KG, KT and GH granted a final approval of the version of the paper to be published and agreed to be accountable for all aspects of the work.

## Conflict of Interest Statement

The authors declare that the research was conducted in the absence of any commercial or financial relationships that could be construed as a potential conflict of interest.

## Footnotes

^1^http://pdsp.med.unc.edu/kidb.php

## Abbreviations

5-CT: 5-carboxamidotryptamine
5-HIAA: 5-hydroxyindoleacetic acid
5-HT: 5-hydroxytryptamine, serotonin
ACSF: artificial cerebrospinal fluid
DRN: dorsal raphe nucleus
SB 269970: (2R)-1-[(3-hydroxyphenyl)sulfonyl]-2-[2-(4-methyl-1-piperidinyl)ethyl] pyrrolidine hydrochloride
sIPSC: spontaneous inhibitory postsynaptic current
WAY 100635: N-[2-[4-(2-methoxyphenyl)-1piperazinyl]ethyl]-N-2-pyridinylcyclohexanecarboxamide.
